# Alternative glacial-interglacial refugia demographic hypotheses tested on *Cephalocereus columna-trajani* (Cactaceae) in the intertropical Mexican drylands

**DOI:** 10.1371/journal.pone.0175905

**Published:** 2017-04-20

**Authors:** Amelia Cornejo-Romero, Carlos Fabián Vargas-Mendoza, Gustavo F. Aguilar-Martínez, Javier Medina-Sánchez, Beatriz Rendón-Aguilar, Pedro Luis Valverde, Jose Alejandro Zavala-Hurtado, Alejandra Serrato, Sombra Rivas-Arancibia, Marco Aurelio Pérez-Hernández, Gerardo López-Ortega, Cecilia Jiménez-Sierra

**Affiliations:** 1 Departamento de Zoología, Escuela Nacional de Ciencias Biológicas, Instituto Politécnico Nacional, CD México, México; 2 Department of Geography, University of Leicester, Leicester, UK; 3 Departamento de Biología, Universidad Autónoma Metropolitana-Iztapalapa, CD México, México; 4 Departamento de Hidrobiología, Universidad Autónoma Metropolitana-Iztapalapa, CD México, México; 5 Escuela de Biología, Benemérita Universidad Autónoma de Puebla, Puebla, México; National Cheng Kung University, TAIWAN

## Abstract

Historic demography changes of plant species adapted to New World arid environments could be consistent with either the Glacial Refugium Hypothesis (GRH), which posits that populations contracted to refuges during the cold-dry glacial and expanded in warm-humid interglacial periods, or with the Interglacial Refugium Hypothesis (IRH), which suggests that populations contracted during interglacials and expanded in glacial times. These contrasting hypotheses are developed in the present study for the giant columnar cactus *Cephalocereus columna-trajani* in the intertropical Mexican drylands where the effects of Late Quaternary climatic changes on phylogeography of cacti remain largely unknown. In order to determine if the historic demography and phylogeographic structure of the species are consistent with either hypothesis, sequences of the chloroplast regions *psbA-trnH* and *trnT-trnL* from 110 individuals from 10 populations comprising the full distribution range of this species were analysed. Standard estimators of genetic diversity and structure were calculated. The historic demography was analysed using a Bayesian approach and the palaeodistribution was derived from ecological niche modelling to determine if, in the arid environments of south-central Mexico, glacial-interglacial cycles drove the genetic divergence and diversification of this species. Results reveal low but statistically significant population differentiation (*F*_*ST*_ = 0.124, P < 0.001), although very clear geographic clusters are not formed. Genetic diversity, haplotype network and Approximate Bayesian Computation (ABC) demographic analyses suggest a population expansion estimated to have taken place in the Last Interglacial (123.04 kya, 95% CI 115.3–130.03). The species palaeodistribution is consistent with the ABC analyses and indicates that the potential area of palaedistribution and climatic suitability were larger during the Last Interglacial and Holocene than in the Last Glacial Maximum. Overall, these results suggest that *C*. *columna-trajani* experienced an expansion following the warm conditions of interglacials, in accordance with the GRH.

## Introduction

Pleistocene climatic oscillations have had a profound effect on the distribution, demographic dynamics and patterns of genetic variation of arid adapted species [1–5]. Cyclic range contractions and expansions shaped the current distribution of the New World of arid plants, their concomitant population dynamics and genetic differentiation, particularly during the Late Quaternary climatic changes [6–7]. However, the influence of these climatic fluctuations on phylogeographic patterns of columnar cacti occurring at the south-central intertropical Mexican drylands remains largely unknown. This region includes the semi-arid communities of the Tehuacán-Cuicatlán Valley (TCV), Río Balsas Basin and Isthmus of Tehuantepec, which is considered the center of origin and diversification of columnar species [8–9]. The TCV harbours the highest diversity and endemism of columnar cacti with 19 species [10]; some of these play a key ecological role because they form dense succulent forests (1,200 individuals ha^-1^), favoring the development of highly diverse communities, which extend over many square kilometers [11]. Thus, conducting phylogeographic studies is essential to better understand the influence of climatic oscillations on the evolution of columnar cacti and the intertropical semi-arid vegetation.

North and South American phylogeographic studies have revealed a pattern of lineage divergence, associated to contraction to and expansion from xerophilous refugia but contrasting responses of species to climatic fluctuation in each hemisphere have been reported [12–16]. In northern Mexico, the genetic signatures of Chihuahuan and Sonoran deserts plants, including the subtropical columnar cacti *Lophocereus schottii*, *Stenocereus gummosus* and *Pachycereus pringlei*, indicate that the genetic divergence is due to population contractions into patches of xerophilous vegetation with warm temperatures during glacials and expansions into different directions during the large-scale aridification phase of the Holocene [17–19]. This demographic dynamic is well supported by palaeoecological analyses of macrofossils from *Neotoma* middens, which point out expansion ranges of floristic elements associated with an increase in aridity during the Holocene interglacial period. Accordingly, xerophytic species persisted in semi-deserts located at low elevations in Sonora and Baja California throughout the last glacial period, from where they reached their current distribution after range expansion at approximately 4 to 8 kyr [20–21]. Theses findings are consistent with the Glacial Refugium Hypothesis (GRH), which is widely accepted for temperate species in the mid latitudes of the Northern Hemisphere. The GRH posits that species contracted to one or more southerly refugia during cold/dry glacial periods and expanded out from them in warm/humidity interglacials [22–25]. Recent Ecological Niche Model (ENM) research on *P*. *pringlei* showed a potential palaeodistribution area restricted to the south of Baja California Peninsula, during the Last Glacial Maximum (LGM ∼21 kya), supporting the GRH. Accordingly, this contraction into southern refugium was followed by a northward colonization during Holocene glacial retreat (10 kya; [26]).

Opposite to this pattern, in the intertropical open dry vegetation of South America (Caatinga, Cerrado and Chaco biomes), populations of some species contracted to warm and humid refugia during the interglacials, and expanded out under the cold/dry climate of the LGM, this pattern is presented as the Interglacial Refugium Hypothesis (IRH; [27–28]). The species complex of the columnar cactus *Pilosocereus aurisetus* seems to validate this hypothesis. The phylogeographic structure of species complex, which inhabit xerophilous scrub enclaves within the Cerrado biome (also known as campo rupestre vegetation), suggests that haplotype divergence is due to isolation events and multiple fragmentations of campo rupestre vegetation during the interglacials and the subsequent expansion and secondary contact among divergent lineages in the glacial period [16]. Range expansions of species complex in the last glacial were consistent with some palynological records, which showed evidence of a drier climate in central and eastern Brazil between 21 to 7 kya [29]. In the same way, the ENM applied to *P*. *aurisetus* complex gave support to IRH and showed a population expansion during the LGM (20 kya), evidenced by a wider and more continuous distribution of species, as well as population fragmentation and retraction to campo rupestre micro-refugia in the warm intervals of the Last Interglacial Period (LIG 135 kya; [16]).

With respect to the south-central intertropical TCV semi-arid zone we do not know what the responses of xerophilous plants had been during this cyclic climatic changes and the lack of reliable fossil records obscure our knowledge of the Quaternary palaeoenvironments. We do not known if regional dry conditions have prevailed since the Mid Miocene [30] and if they became more acute during Quaternary glacial-interglacial cycles. To address these gaps, the present study used chloroplast DNA sequences and distribution species model of *Cephalocereus columna-trajani* (kya) K. Schum., in order to infer the impact of Quaternary climatic fluctuations on the phylogeographic pattern of columnars within this region. It is estimated that the age of origin for *C*. *columna-trajani* is 2.65 Mya (95% HPD, 1.36–4.23; [31]) and was probably derived from an Isthmian ancestor of the subgenus *Neodawsonia* through geographic isolation in TCV during the Pliocene/Pleistocene (5.33–2.58 Mya; [32]). Thus, the species represents an appropriate system to infer for the first time the response and genetic signals of Late Quaternary climatic changes on cacti from the intertropical North America semi-arid zones.

In this work we proposed to contrast the alternative scenarios of the Glacial Refugia Hypothesis and Intergalcial Refugia Hypothesis to specifically infer whether historical demography dynamic and genetic divergence of the species matches to either of scenarios. According to the GRH, if *Cephalocereus columna-trajani* experienced a contraction during the glacial period, a decrease in palaeodistribution area and effective population size would be detected at some point in this period, while population and spatial size would increase during one of the interglacial periods. However, if the demographic dynamics of *C*. *columna-trajani* would match the IRH, higher population size, and palaeodistribution area would be revealed during the Last Glacial Maximum, while during the interglacials a reduction in population size and area of palaeodistribution can be inferred. Using a molecular Bayesian analysis and ecological niche model, the present work aimed to: (1) evaluate the diversity and phylogeographic structure of *C*. *columna-trajani*; (2) determine the sequence and magnitude of Late Quaternary climatic changes in effective population size to contrast GRH and IRH demographic hypotheses; and (3) analyse the predicted current and past potential distribution areas of the species in order to infer if Late Quaternary climatic changes modified its distribution range and population connectivity.

## Materials and methods

### Species and study site

*Cephalocereus columna-trajani* has a discontinuous distribution throughout the TCV and is restricted to hillsides with pronounced slopes at elevations of 800–1800 m asl [33–35]. This species is the dominant element of the xerophytic communities known as *organales* (470–540 plants ha^-1^, [36]), but it can also form part of the seasonally dry tropical forest [37].

**Fig 1:**
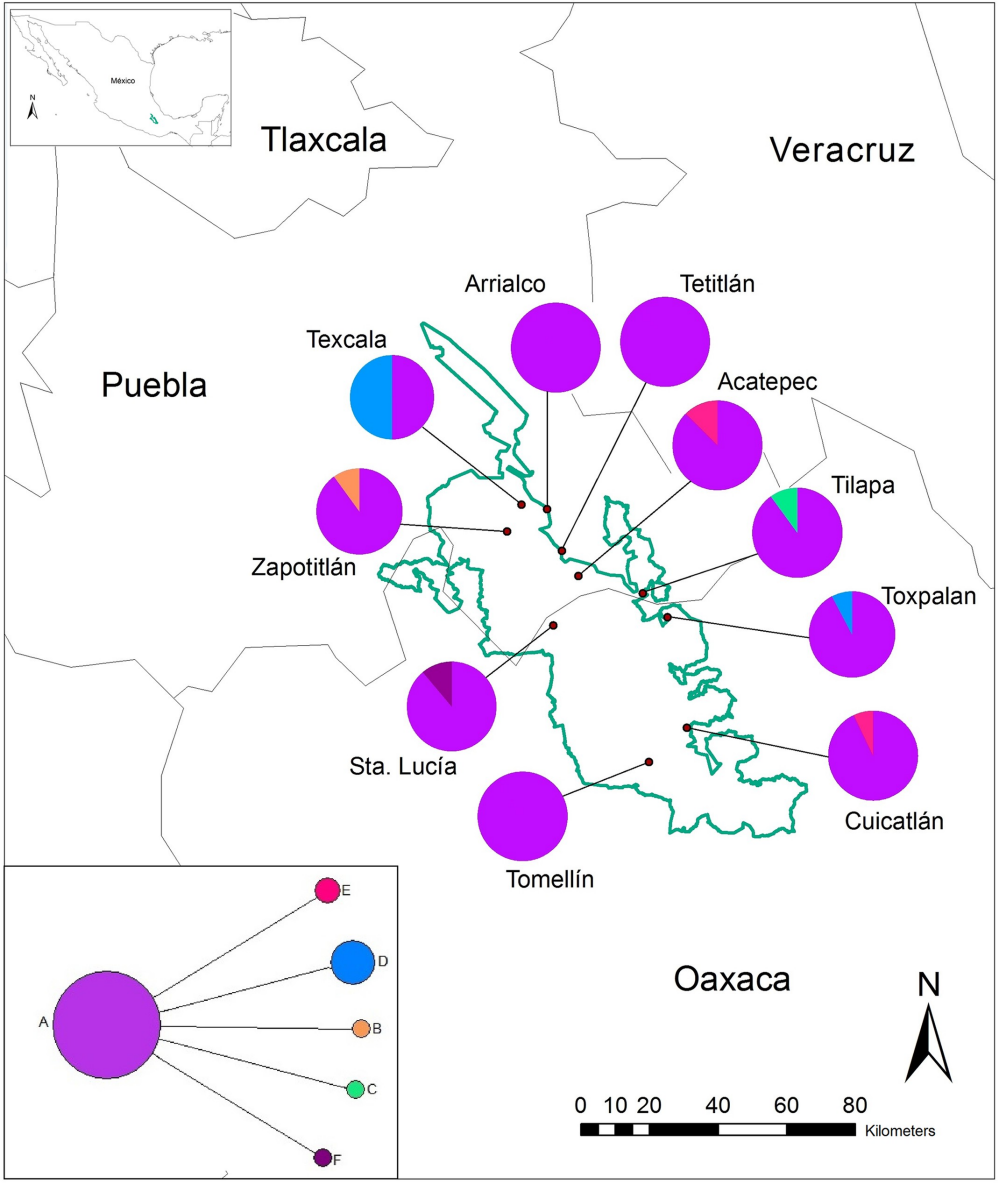
Geographic distribution and statistical parsimony network (lower left insert) of the six haplotypes of *Cephalocereus columna-trajani* from the Tehuacán-Cuicatlán Valley. Pie graphs show the frequency of haplotypes in each population. The solid line denotes the boundaries of the Tehuacán-Cuicatlán Biosphere Reserve.

The study was carried out within the Tehuacan-Cuicatlan Biosphere Reserve, located in the south-eastern part of the state of Puebla and the northwest of Oaxaca between 17°39’ and 18°53’ N, and 96°55’ and 97°44’ W (Fig 1). The TCV is characterized by semiarid climate, BS_o_hw(w) with summer rains, a mean annual precipitation of 443.7 mm, and a temperature of 17.9°C [38].

### Tissue collection, DNA extraction and sequencing

Photosynthetic tissue was collected from 140 randomly selected individuals in 10 localities comprising the full distribution range of the species (Table 1). The tissue was transported in a liquid nitrogen thermos and transferred to an ultra-low freezer at -80°C until DNA extraction. Approximately 200 mg of chlorenchyma from 110 individuals were used, in accordance with the protocol of the DNEasy Plant Mini Kit (Qiagen, Hilden, Germany).

**Table 1:** Collection localities and standard descriptors of genetic diversity in ten populations of *Cephalocereus columna-trajani* from the Tehuacán-Cuicatlán Valley. Numbers in parentheses are the standard error.

Population	N	Geographic Coordinates	Altitude (m asl)	*h*	*π*
**Texcala**	10	18°23'N, 97°26’W	1714	0.556 (0.0056)	0.00064 (0.00)
**Arrialco**	11	18°23’N, 97°22’W	1422	0.000 (0.000)	0.000 (0.000)
**Zapotitlán**	10	18°19’N, 97°28'W	1500	0.200 (0.0238)	0.00023 (0.00)
**Tetitlán**	13	18°16’N, 97°19'W	1194	0.000 (0.000)	0.000 (0.000)
**Acatepec**	8	18°12’N, 97°17'W	1174	0.250 (0.0324)	0.00029 (0.00)
**Tilapa**	10	18°10’N, 97°07'W	861	0.200 (0.0238)	0.00023 (0.00)
**Toxpalan**	13	18°06’N, 97°03'W	1225	0.154 (0.0159)	0.00018 (0.00)
**Santa Lucía**	9	18°04’N, 97°21'W	1793	0.222 (0.0276)	0.00026 (0.00)
**Cuicatlán**	14	17°48’N, 97°00'W	1110	0.143 (0.0141)	0.00016 (0.00)
**Tomellín**	12	17°43’N, 97°06'W	1110	0.000 (0.000)	0.000 (0.000)
**Total**	110			0.188 (0.0024)	0.00023 (0.0000)

*h*, haplotype diversity; *π*, nucleotide diversity.

The chloroplast regions *psbA-trnH* and *trnT-trnL* were amplified. In the former, the primers of Butterworth and Wallace [39] and Sang *et al*. [40] were used. PCR was carried out in a total volume of 25 μl containing 2.5 μl of 10X PCR buffer (New England BioLabs, Ipswich, MA), 0.75 μl MgCl_2_ (50 mM), 0.5 μl of an equimolar solution of dNTP’s (10 mM), 0.2 ml of each primer (14 pmol), 0.5 μl Taq polymerase (5 U/μl) and 10 ng/μl of template DNA. The amplification program consisted of an initial denaturation step at 94°C (2 min), followed by 25 cycles of 94°C (1 min), 50°C (2 min), and 72°C (1 min), and a final extension step at 72°C for 10 min. Amplification conditions for the *trnT-trnL* region were performed as described above except for the MgCl_2_ volume, which was 0.875 μl. The primers B48557-F and B49291-R were used [41]. This amplification program was as follows: preheating at 94°C (2 min), followed by 25 cycles of 94°C (1 min), 55°C (1 min) and 72°C (1 min), and a final extension step at 72°C for 15 min. The amplification products were purified using the QIAquick PCR Purification Kit (Qiagen, Valencia, CA) and sequenced with BigDye Terminator v3.1. Sequenced products were analysed in an ABI Prism 3100-Avant Genetic Analyser. Finally, the sequences were manually aligned and edited with SEQUENCHER v4.8 software (Gene Codes Corporation, Ann Arbor, MI, USA). The sequences were deposited in the NCBI GenBank database (Accession Numbers KY363333-KY363338).

### Data analysis

#### Relationships between haplotypes

Based on the two concatenated sequences, the haplotypes of the species were determined using DNASP v5.10 [42]. The genealogical relationships among these haplotypes were subsequently inferred and the most parsimonious network was constructed using the median-joining (MJ) algorithm with NETWORK v4.6.1.1 (http://www.fluxus-engineering.com). Using the number of different nucleotides between two sequences, the genetic distance between them was estimated.

#### Genetic structure and diversity

Haplotype diversity (*h*) and nucleotide diversity (*π*) were estimated at the species and population level using DNASP v5.10 [42–43]. Genetic differentiation was calculated by analysis of molecular variance (AMOVA; [44]), using paired differences and haplotype frequencies with ARLEQUIN v3.5 [45]. Estimates of haplotype divergence between populations (*F*_*ST*_) were calculated and their significance was obtained using 1,000 nonparametric permutations [44]. Also, genetic groups were identified with a Bayesian analysis for assigning individuals, using STRUCTURE v2.3 [46]. The admixture model was selected, with correlated allele frequencies and location sampling as prior information; default values were used for all other parameters. Each run consisted in 10,000 burn-in iterations and 100,000 iterations. A total of 15 runs were performed for each *K* cluster (*K* = 1 to 10). The most probable number of clusters was estimated by calculating the natural logarithm of the likelihood function [47], and the *ΔK* statistic was estimated as described in Evanno *et al*., [48]. A consensus Q-matrix was constructed from the 20 runs with CLUMPP v1.1.2 [49] and the homogeneous groups of plants were then visualized with DISTRUCT v1.1 [50].

#### Demographic history

The demographic history of *Cephalocereus columna-trajani* was inferred by means of Bayesian Skyline Plot (BSP) analysis, which correlates the magnitude and point in time at which changes in effective population size (*N*_*e*_) took place, using BEAST v1.6.1 [51]. For both regions, were used the HKY substitution model, the uncorrelated lognormal relaxed-clock model, an estimated nucleotide substitution rate of *μ* = 1×10^−9^ to 3×10^−9^ neutral substitutions per site per million years [52], and constant population size as priors. The MCMC chain replicates were made in 10 million steps with 10% burn-in, while trees and parameters were sampled every 1000 steps. All other parameters were specified with default values. The *N*_*e*_*μ* values and confidence intervals were estimated with TRACER v1.5.0 based on probability distributions and were synthesized as a BSP [53].

The demographic dynamic of *Cephalocereus columna-trajani* population was analysed by Approximate Bayesian Computation (ABC), using DIYABC v2.03 program [54]. Mutually exclusive demographic scenarios were made to compete under the GRH and IRH, and the scenario with the highest posterior probability was identified. If the GRH applies, *N*_*e*_ would be expected to increase during either to LIG or Holocene, while in the IRH it would increase along the glacial period. All ten populations were clustered into a single one and seven competing demographic scenarios (based on a combination of demographic events including bottleneck, population expansion, reduction, and constant population size) were evaluated (S1 Fig). Minimum and maximum values were established for demographic events as well as for parameters in the priors, assuming a unimodal distribution (S1 Table). The HKY mutation model and a mutation rate of 1×10^−9^ to 3×10^−9^ were used for both sequences [52]. The reference table consisted of one million pods (pseudo-observed data sets) per scenario and per set of priors. The scenarios were compared by calculating their relative posterior probability by polychotomous logistic regression on 0.1% of the simulated data sets closest to the observed data sets [54]. Once the most probable scenario had been identified, the posterior distributions of parameters were estimated by logit transformation of parameters and linear regression on 0.1% of the simulated data sets closest to the observed data sets. To assess the confidence level of the most probable scenario, new data sets were simulated for each scenario in order to estimate their relative probability and the percentage of times that the correct scenario has the highest posterior probability of occurring [55]. Based on these results, type I and type II errors were estimated for the selected scenario. A type I error is the probability of excluding the selected scenario when it is the correct one, and a type II error is the probability of choosing the selected scenario when it is not the correct one. Finally, the goodness of fit of the best scenario was evaluated using the model checking option [55]. Consistency between parameter priors and the predictive posterior distribution of the scenario was assessed. For goodness of fit of the model to be considered adequate, observed statistics must fall within the distributions of simulated statistics [55].

#### Palaeodistribution modelling

The GRH implies that palaeodistribution of *Cephalocereus columna-trajani* populations was interrupted during LGM while under interglacials they were continuous. The opposite pattern would be expected if the species responded according to the IRH. These hypotheses were evaluated using Ecological Niche Model. For this, 37 presence records of *C*. *columna-trajani* were compiled. These were obtained directly in the field (Garmin 60CS GPS) or from the main national herbariums (MEXU, ENCB, IZTA) as well as from the biological collections of the World Information Network on Biodiversity (http://www.conabio.gob.mx) and Global Biodiversity Information Facility (http://www.gbif.org). Similarly, the layers of 19 climatic variables, based on general circulation model (GCM) simulations of the Community Climate System Model (CCSM-4; [56–57]), with a spatial resolution of 2.5 min (approximately 4.5 Km/pixel) were obtained from the WorldClim website (http://www.worldclim.org). The area of interest, the M area, was determined by the intersection of terrestrial ecoregion polygons [58] with the 30 Km buffer area were established on the basis of presence records. Variables were selected by principal components analysis (PCA) to remove correlated variables and to prevent model over adjustment (Annual Temperature, Isothermality, Minimum Temperature of Coldest Month, Temperature Annual Range, and Mean Temperature of Coldest Quarter; S2 Table). Using presence data and the selected climatic variables, the current potential distribution was modelled with the maximum entropy algorithm in MAXENT v3.2.2 [59–60], using the standard configuration and 25% of the presence data for model training. The model was validated calculating the area under the curve generated by the receiver operating characteristic curve (AUROCC; [61]). Based on the model of the current potential distribution as well as the palaeoclimatic variables obtained from CCSM-4 glacial simulation (http://www.worldclim.org) with a resolution of 4.5 km, the potential distributions of *C*. *columna-trajani* in the Middle Holocene (MH, 6 kyr), the LGM (22 kyr) and the LIG (135 kyr) were predicted. Finally, changes in the suitability of the predicted distribution area for each of these periods were compared with the current potential distribution area. Additionally, a comparison of climatic variables was done with a MANOVA.

## Results

### Relationships between haplotypes

The concatenated data sets of the sequences *psbA-trnH* and *trnT-trnL* (867 bp) produced six haplotypes. The distribution and frequency of these haplotypes are shown in Fig 1. The statistical parsimony network revealed a high frequency haplotype *A*, which was recovered from all ten populations of *Cephalocereus columna-trajani* (Fig 1). The haplotypes *B*, *C* and *F*, connected to *A* by one step, are restricted to the Zapotitlán, Tilapa and Santa Lucía populations, respectively. Haplotype *D* was recovered in Texcala and Toxpalan, and haplotype *E* in Acatepec and Cuicatlán populations. The haplotype network shows a star-like genealogy, where the internal position and large number of connections of haplotype *A*, as well as its wide distribution, suggest that this haplotype represents an ancestral condition.

### Genetic diversity and structure

Nucleotide diversity in the 110 sequenced individuals showed a low mean value (*π* = 0.00023). Texcala was the population with the highest value (*π* = 0.00029) while the lowest one was obtained in Arrialco, Tetitlán and Tomellín, (*π* = 0.00000; Table 1). The mean haplotype diversity was *h* = 0.188. The lowest values occurred in Arrialco, Tetitlán and Tomellín (*h* = 0), and the highest in Texcala (*h* = 0.556, Table 1). The AMOVA revealed that the greater part of the genetic variation is found within populations, and differences among populations are low but statistically significant (*F*_*ST*_ = 0.124, P < 0.001; Table 2). The Bayesian analysis to assigning individuals showed three significant genetic clusters (*ΔK* = 3) with no clear geographic structure (Fig A and B in S2 Fig). Based on this result, the ten *Cephalocereus columna-trajani* populations were considered a single panmictic population in all subsequent ABC analyses.

**Table 2:** Analysis of molecular variance (AMOVA) based on the regions *psbA-trnH* and *trnT-trnL* from *Cephalocereus columna-trajani*. P < 0.001.

Source of variation	df	Sum of squares	Variance components	Percent variation
**Between populations**	9	4.497	0.027	12.443
**Within populations**	100	19.53	0.195	87.556
**Total**	109	24.027	0.223	
**Fixation index**	0.124			

### Demographic history

The demographic *ABC* analysis results supported species population expansion during the LIG period with a posterior probability equal to *PP* = 0.318 (IC 95% 0.299–0.338), which were included within GRH competing scenarios (Scenario 3; Table 3, S1 Fig). The type I error indicates that the probability of excluding the best scenario (population expansion during the LIG period) when it is the correct one is low (0.106), and the type II error indicates that the probability of selecting the best scenario when it is not the correct one is also relatively low (0.164; Table 3). Based on this scenario, the expansion occurs at 123.04 kya (95% CI 115.3–130.03; Table 4). The estimated population size of the *Cephalocereus columna-trajani* before the expansion was *Nb* = 996,000 individuals (95% CI 327,000–1,840,000), increasing up to an estimated size of *N1* = 1,680,000 individuals (95% CI 702,000–2,370,000).

**Table 3:** Description of Glacial and Interglacial Refugia hypothetical scenarios of *Cephalocereus columna-trajani* that were compared using the ABC approach. The posterior probability (*PP*), 95% credible interval (95% CI), and type errors I and II are shown. The most probable scenario is highlighted in bold letters.

Analysis	Scenarios description	PP [95% CI]	Error Type I	Error Type II
**Glacial Refugia hypothesis**	1. Holocene expansion	0.2864 [0.2649–0.3078]	0.0693	0.1280
	2. Last Glacial Maximum bottleneck	0.0484 [0.0375–0.0594]	0.1300	0.1173
	**3. Last Interglacial expansion**	**0.3183** **[0.2987–0.3379]**	**0.1067**	**0.1640**
**Interglacial Refugia hypothesis**	4. Holocene reduction	0.0593 [0.0466–0.0719]	0.0983	0.1633
	5. Last Glacial Maximum expansion	0.0493 [0.0370–0.0616]	0.1147	0.1607
	6. Last Interglacial reduction	0.0837 [0.0711–0.0962]	0.1470	0.0880
**Null hypothesis**	7. Constant population size	0.1546 [0.1390–0.1703]	0.1653	0.0100

**Table 4:** Estimates of the posterior distribution of the demographic parameters revealed by ABC analysis for the Scenario 3: Last Interglacial expansion of *Cephalocereus columna-trajani*.

Parameter	Mean	Median	Mode	95% CI
***t***_***3***_	123.04	123.04	130.03	115.3–130.03
***Nb***	996000	950000	806000	327000–1840000
***N1***	1680000	1730000	2140000	702000–2370000

*t*_*i*_ is expressed in kyr and was computed assuming a mean time of 69.91 years to first reproduction in the species [36]; *Nb*: population size at reduction; *N1*: current population size; CI: credible interval.

**Fig 2:**
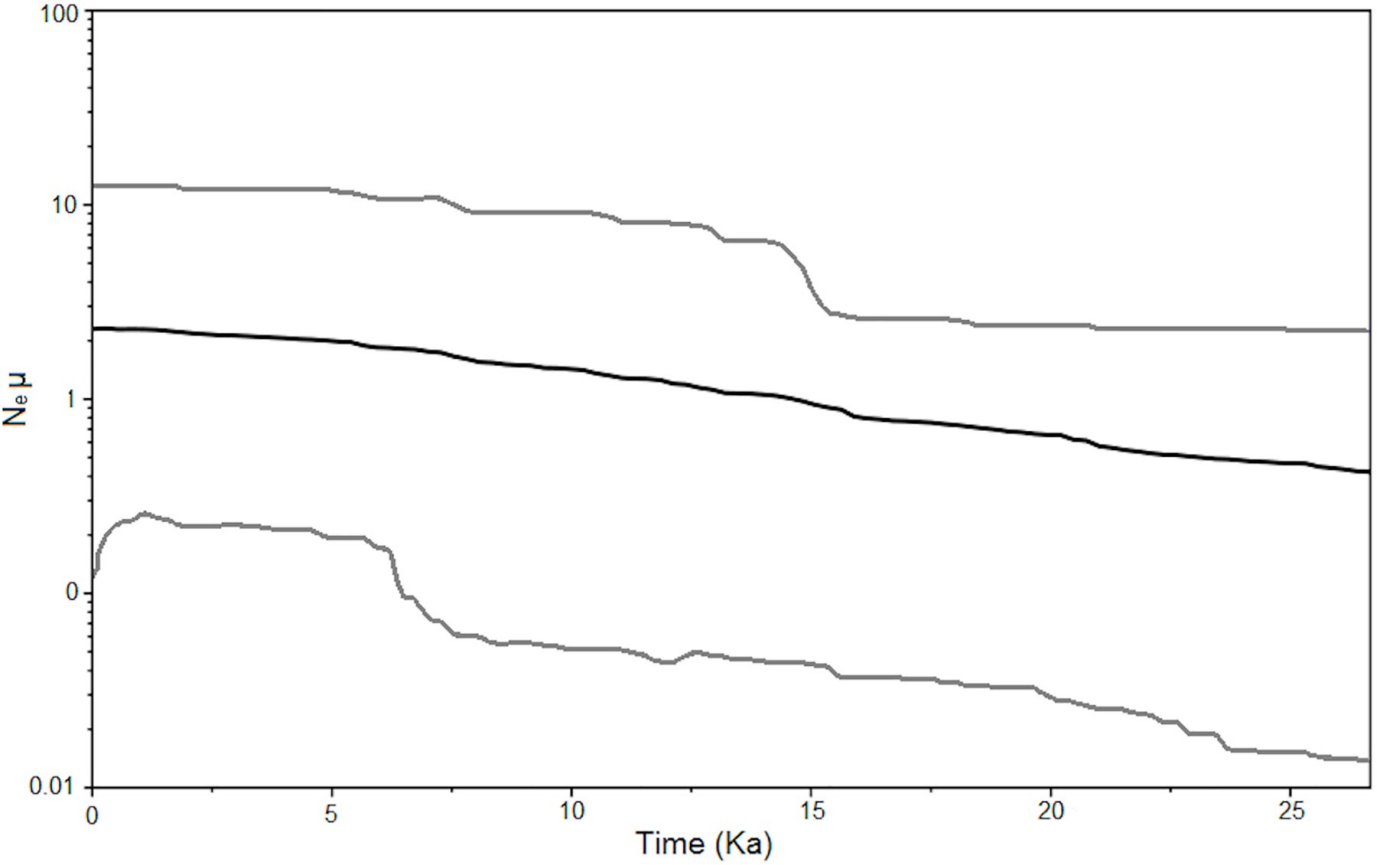
Late Quaternary demographic trend of *Cephalocereus columna-trajani*, obtained by Bayesian Skyline Plot analysis. The black line represents the mean and the gray lines the limits of the 95% HPD (Highest Posterior Density).

According to the BSP analysis, the effective population size of *Cephalocerus columna-trajani* was maintained relatively stable during the LGM, but at the end of the last glacial stage (14.5 kya), when ice sheets started to melt, population size began to increase and continued throughout the Holocene (Fig 2).

### Palaeodistribution modeling

**Fig 3:**
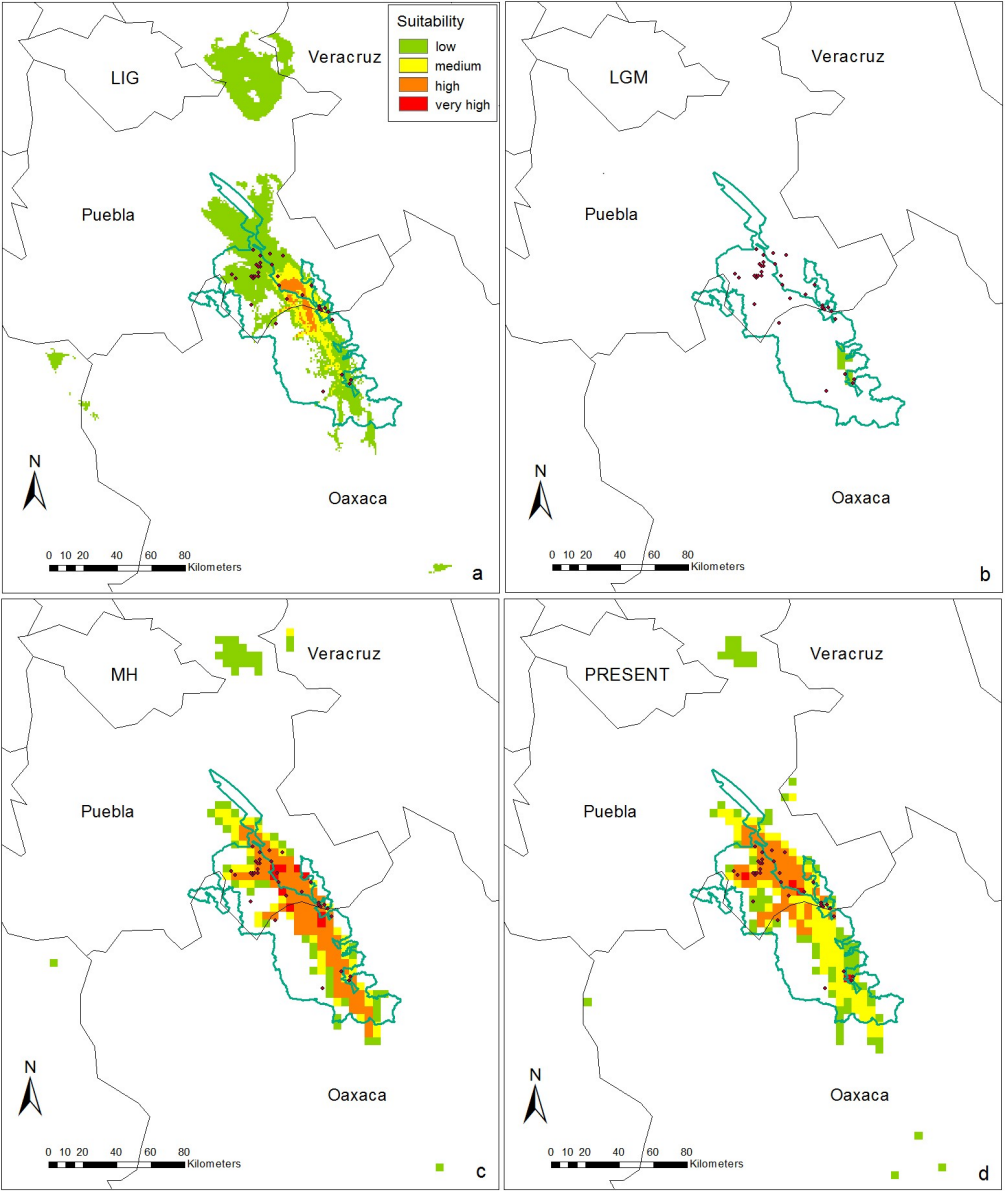
Distribution models and suitability index of *Cephalocereus columna-trajani*. (A) Last Interglacial (LI; 122 Kyr), (B) Last Glacial Maximum (LGM; 22 Kyr), (C) Middle Holocene (HM; 6 Kyr), and (D) Present distribution obtained with MAXENT v3.2.2 [60]. Suitability refers to environmental characteristics where a species stay and reproduce in a specific site. Low values indicate unsuitable climatic conditions for species presence; high values indicate more suitable conditions for growth of the species.

The ecological niche model generated for *Cephalocereus columna-trajani* showed good performance with the data used (AUC = 0.908). Likewise it suggests that the changes in potential distribution over time are consistent with the GRH, i.e. the species found more suitable climatic conditions during the interglacials, supporting to the preview selected *ABC* demographic scenario. During the warm climatic conditions of the LIG the palaeodistribution area was the widest than at any other time period (1,810 km^2^), although moderate or low suitability for the species were shown (Fig 3A). In contrast, during the cold and dry LGM, the potential palaeodistribution area of *C*. *columna-trajani* became considerably reduced in relation to other epochs and revealed lowest climatic conditions suitability within the TCV (27 km^2^, Fig 3B). The potential palaeodistribution area of MH is similar to the current one (931.5 and 999 km^2^, respectively), but very high and high suitabilities represent a larger percentage in the MH than the current one distribution where moderate and low suitability areas predominate (Fig 3C and 3D). According to a MANOVA analysis based on the 19 climatic variables the conditions within the TCV had fluctuated significantly throughout the Quaternary (Table A and B in S3 Table).

## Discussion

The pattern of genetic variation, phylogeographic structure and historic demography suggest that the intertropical columnar cacti *Cephalocereus columna-trajani* experienced a population expansion across the TCV during the warmer periods of the Late Quaternary, a pattern that is consistent with the GRH and validated by palaeodistribution modelling.

The low genetic diversity and excess of rare alleles, as well as the widespread distribution of haplotype *A* in all populations, reflected an expansion by long distance dispersal [23, 61], related to the colonization of isolated populations with very little or no genetic variation [62]. Within the TCV, the long distance dispersal of columnar cacti is performed by phyllostomids bats, such as *Choeronycteris mexicana* and *Leptonycteris nivalis*, which move a low proportion of seeds within their gut during feeding periods [63–65]. Considering that bats can move up to 30 Km during one night to their foraging areas [66], it is likely that they disperse seeds far away from maternal plants [64]. Thus long-distance seed dispersal by bats could have played a key role in the expansion of *Cephalocereus columna-trajani*, as well as in the formation of isolated and low diversity populations due to recurrent bottleneck.

The genetic structure obtained by means of AMOVA and STRUCUTRE revealed low, but statistically significant population differentiation in *Cephalocereus columna-trajani* (*F*_*ST*_ = 0.124, P < 0.001), with no clear geographic pattern, which may be due to the influence of genetic drift associated with random colonization [67]. The genetic differentiation could be explained by another aspect of the feeding behaviour of *Leptonycteris curasoe* and *Choeronycteris mexicana*, which could favour short distance seed dispersal. After removing a piece of fruit, bats immediately perch on some perennial nurse shrubs, where they consume the pulp and spit out the majority of seeds, dropping them to the ground near the maternal plant [64]. This behaviour can form narrow genetic neighbourhoods, promoting certain degree of inbreeding and the interpopulation differentiation within the discontinuous habitat of *Cephalocereus columna-trajani*. Similar levels of genetic differentiation were found in populations of South American columnars with chiropterophillous pollination sindrome like *Stenocereus griseus* and *Cereus repandus* (*G*_*ST*_ = 0.092 and 0.126 respectively; [68]), or else higher levels as in *Praecereus euchlorus* (*θ* = 0.484; [69]).

### Demographic history and palaeodistribution

*Cephalocereus columna-trajani* showed similar responses to Late Quaternary climatic fluctuations to other subtropical North American arid plants, including cactuses expanding during the warmer climatic conditions of the interglacial periods [70]. Based on the ABC demographic analyses, population expansion of *C*. *columna-trajani* corresponds chronologically to the LIG (*N1* = 1,680,000 individuals; 95% CI 702,000–2,370,000 and 123.04 kyr; 95% CI 130.03–115.3) and, according to the BSP analyses, to the Holocene (Fig 2). In the current interglacial, the climatic conditions that favour the population growth of Sonoran Desert *Carnegia gigantea* include frost-free winters and monsoonal summer rainfall, which foster regeneration and adult survival [71–72]. In contrast recurring frost (temperatures between -8.3°C and -5.6°C, lasting between 15 to 20 hours) significantly reduce seedling establishment and increase the mortality of reproductive adults, having catastrophic consequences on population sizes [71–72]. It is likely than during the Quaternary cooling periods, a high frequency of frosts and reduced rainfall negatively affected the population growth of subtropical cacti as well as species with similar adaptations to environmental conditions [12]. Within the TCV, and according to WorldClim data, the LIG and HM were warmer and wetter than the LGM; suggesting more suitable climatic conditions that could favour the population growth of *C*. *columna-trajani* during interglacials. In contrast, during the last glacial period more severe aridity prevailed, so it is possible that the frequency and duration of frosts were increased, reducing growth and survival rates of *C*. *columna-trajani* what adversely affected the population size [*Nb* = 996 000 individuals; 95% CI 327,000–1,840,000].

Consistent with ABC results, Ecological Niche Model predictions revealed a largest areas of potential palaeodistribution of the species under the climatic conditions of the LIG and the MH; than during the LGM whose palaeodistribution was reduced to the minimum (Fig 3B). In the same way, the palaeodistribution of *Pachycereus pringlei* during the LGM was restricted to a southern reduced area of the Baja California Penninsula, which could have acted as a refugium [26]. The LIG was warmer than the Holocene and the latter was warmer than the present [73–74], which may explain why a larger palaeodistribution area and higher suitability were predicted for these periods, particularly in the MH.

According to the expansions within the TCV, it might be possible that *Cephalocereus columna-trajani* attained its modern range distribution by a rapid colonization from south to north after the LGM. The highest present day habitat suitability (Fig 3D) and density of individuals (540 individuals ha^-1^ in Tetitlán; [36]), as well as the higher richness of columnar cacti are located in the north of the TCV where arid environments predominate [33]. However this colonization hypothesis needs to be tested by mean of nuclear markers studies. It is also possible that *C*. *columna-trajani* experienced an altitudinal expansion within the complex topography of the TCV [75]. Species of *Bursera*, the dominant trees of seasonally dry tropical forest of Mexico, not only expanded their geographic range after de LGM, but also experienced an important elevational gradient distribution during the current interglacial [76]. The range expansion of *Bursera* species seemed to be a response to climate warming in its heterogeneous topography in the area of distribution, which include the TCV. Alternatively, since the TCV is a stripe area surrounding by mountain ranges, which has remained orographically isolated as a relatively stable arid habitat [10], it is possible that throughout glacial-interglacial cycles *C*. *columna-trajani* populations persisted *in situ* [77].

The interaction between *Glossophagine* members and columnar cacti seems to be ancient; it was probably established since the Miocene [31]. Thus it is likely that the colonization of columnar cacti in the intertropical Mexican dry lands could have historically facilitated by this interaction. In conjunction, the biotic interaction described above and climate-imposed conditions could have been determining factors in the expansion of *Cephalocereus columna-tajani*. The phylogeographic pattern of *C*. *columna-trajani* could had been affected by cycle climatic changes in a similar way as had occurred in *Vitellaria paradoxa*, the karate tree of the tropical African savanna, whose phylogeografic structure was determined by the fragmentation of populations during the dryer and colder conditions of the LGM [3].

Palynological records of the Middle Miocene Tehuacan Formation (15–17 Mya) suggest a change from a relatively humid to a semiarid climate, which favoured the expansion of groups currently abundant in the xerophilous scrub and low-growth seasonally dry tropical forest such as Cactaceae, Agavaceae, Burseraceae, Leguminosae, Compositae, Graminaceae and *Ephedra* [30]. The present study suggests for the first time that if during Late Quaternary severe arid conditions persisted at LGM, they could determine the distribution, demographic dynamics and genetic differentiation of *Cephalocereus columna-trajani*, one of the most abundant columnar within the TCV. Future studies need to be carried out on other ecologically important columnar cacti from this region, including species that are co-distributed with *C*. *columna-trajani* and display chiropterophilous pollination and seed dispersal modes such as *Neobuxbaumia tetetzo*, *Pachycereus weberi*, *Pilosocereus chrysacanthus* and *Pseudomitrocereus fulviceps* to determine whether the response of these species was consistent with the Glacial Refugia Hypothesis and if a generalised climate change pattern can be established.

## Supporting information

S1 Fig Diagrams of the competing demographic scenarios of *Cephalocereus columna-trajani*. The Glacial Refugia Hypothesis (Scenario 1: Holocene expansion, Scenario 2: Last Glacial Maximum bottleneck; Scenario 3: Last Interglacial expansion), Interglacial Refugia Hypothesis (Scenario 4: Holocene reduction; Scenario 5: Last Glacial Maximum expansion; Scenario 6: Last Interglacial reduction) and null scenario (Scenario 7: Constant population size). Effective population size is shown in different colours and the time of occurrence of events is expressed in generations ago. *NA*: ancestral population, *N1*: current population, *Na*: population at expansion, *Nb*: population at reduction, *NB*: population at bottleneck; *t*_*i*_: time of an event; *DB*: duration of bottleneck; *DE*: duration of expansion. The values used for these parameters are listed in Table S1.
(TIF)

S2 Fig Genetic clustering of *Cephalocereus columna-trajani* according to STRUCTURE analysis. (A) Clustering of individuals at the most likely *K* value (*K* = 3). Each vertical line represents an individual, each colour represents a cluster, and black lines separate individuals from different populations. (B) Estimation of the most likely number of clusters (*ΔK*).
(TIFF)

S1 Table Prior distributions of demographic and historic parameters, and the set conditions used in the ABC analyses to test the Glacial and Interglacial Refugia Hypotheses on *Cephalocereus columna-trajani*. (DOCX)

S2 Table Principal component analysis of climatic variables. Loadings of each variable in the first three principal components (PC_i_). Bold shown most important variables.
(DOCX)

S3 Table Comparison of climatic variables among the four periods analysed. A) Values of different estimator in MANOVA. After obtaining a significant MANOVA, we test each variable with an ANOVA, and all bioclimatic condition were statistically different among all periods of time. *F* test value, *DF1* = degree of freedom among groups; *DF2* = degree of freedom of error; *P*-value.
(DOCX)
