# Supplementary figures and images for: Alternative glacial-interglacial refugia demographic hypotheses tested on Cephalocereus columna-trajani (Cactaceae) in the intertropical Mexican drylands
Source: PLoS One. 2017 Apr 20;12(4):e0175905. doi: 10.1371/journal.pone.0175905 (PMC5398652; doi:10.1371/journal.pone.0175905)

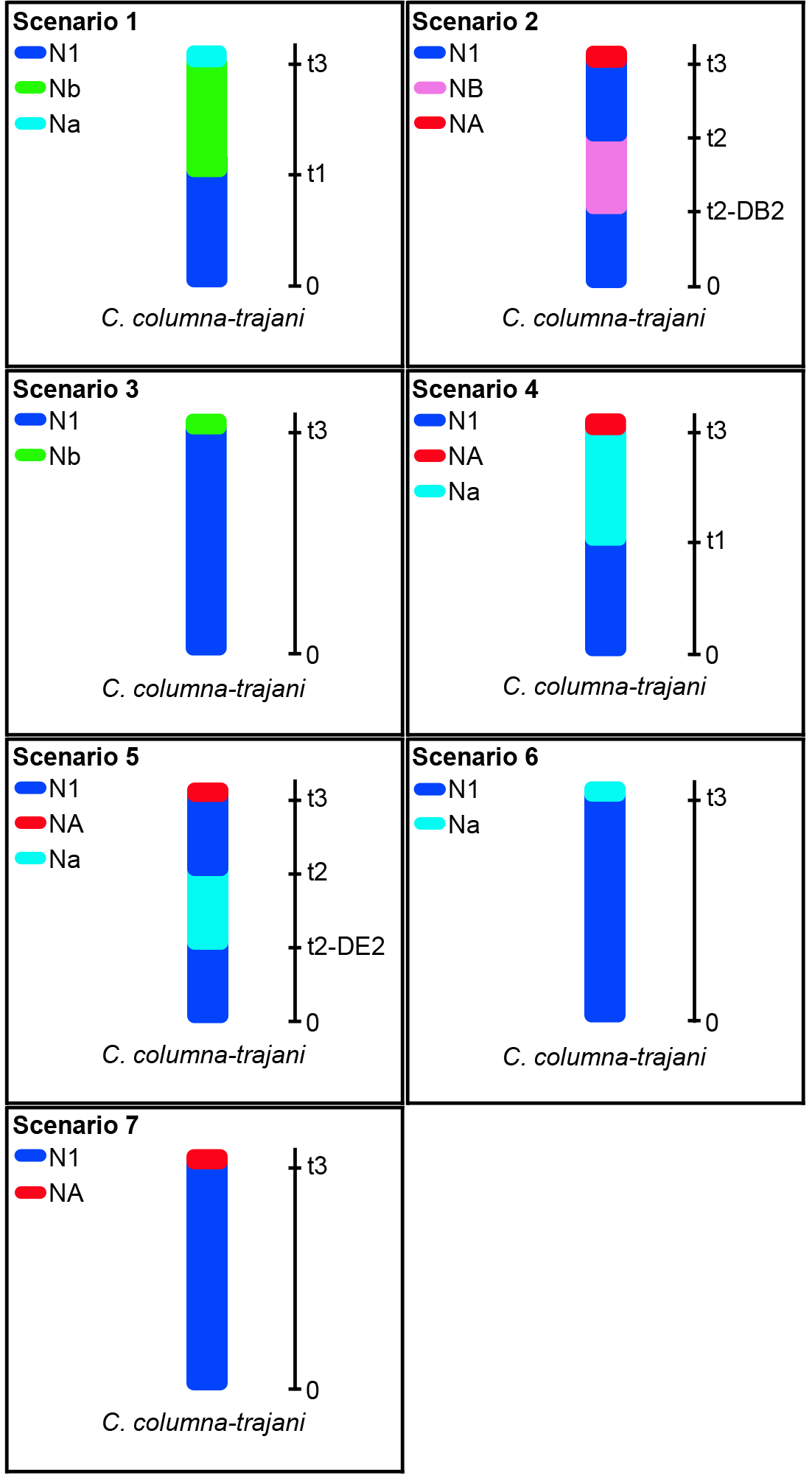

Supplement: S1 Fig — The Glacial Refugia Hypothesis (Scenario 1: Holocene expansion, Scenario 2: Last Glacial Maximum bottleneck; Scenario 3: Last Interglacial expansion), Interglacial Refugia Hypothesis (Scenario 4: Holocene reduction; Scenario 5: Last Glacial Maximum expansion; Scenario 6: Last Interglacial reduction) and null scenario (Scenario 7: Constant population size). Effective population size is shown in different colours and the time of occurrence of events is expressed in generations ago. NA: ancestral population, N1: current population, Na: population at expansion, Nb: population at reduction, NB: population at bottleneck; ti: time of an event; DB: duration of bottleneck; DE: duration of expansion. The values used for these parameters are listed in Table S1. (TIF) [file pone.0175905.s001.tif]

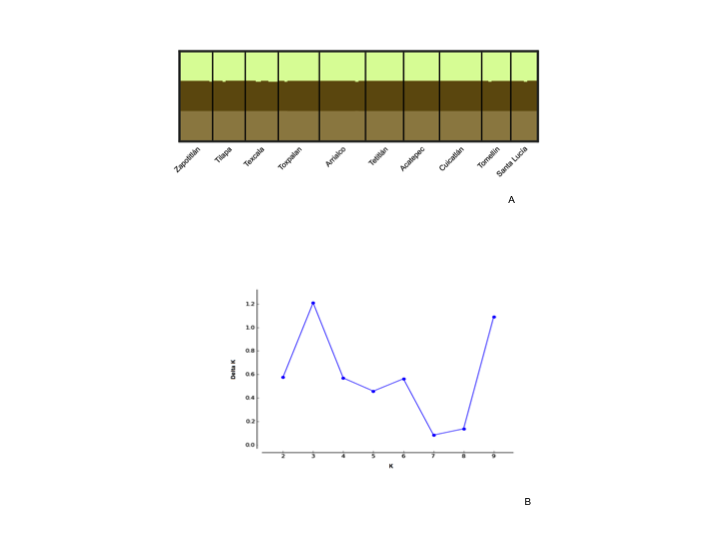

Supplement: S2 Fig — (A) Clustering of individuals at the most likely K value (K = 3). Each vertical line represents an individual, each colour represents a cluster, and black lines separate individuals from different populations. (B) Estimation of the most likely number of clusters (ΔK). (TIFF) [file pone.0175905.s002.tiff]
