# Supplementary material for: Alternative glacial-interglacial refugia demographic hypotheses tested on Cephalocereus columna-trajani (Cactaceae) in the intertropical Mexican drylands
Source: PLoS One. 2017 Apr 20;12(4):e0175905. doi: 10.1371/journal.pone.0175905 (PMC5398652; doi:10.1371/journal.pone.0175905)
Supplement: S1 Table — (DOCX) [file pone.0175905.s003.docx]

**S1 Table. Prior distributions of demographic and historic parameters, and the set conditions used in the ABC analyses to test the Glacial and Interglacial Refugia Hypotheses on *Cephalocereus columna-trajani*.**

| Parameters | Min. | Max. |
| --- | --- | --- |
| *NA* | 120000 | 4800000 |
| *N1* | 240000 | 2400000 |
| *Na* | 480000 | 4800000 |
| *Nb* | 200000 | 2000000 |
| *NB* | 12000 | 120000 |
| *t_1_* | 1 | 172 |
| *t_2_* | 173 | 1016 |
| *t_3_* | 1645 | 1860 |
| *DB2* | 35 | 350 |
| *DE2* | 75 | 750 |
| *NA≤Nb, NA≥Na, Na>N1, Nb<N1, NB<Nb, t_3_>t_2_, t_2_>t_1_* | | |

*Ni* = population size (*NA*: ancestral population, *N1*: current population, *Na*: population at expansion, *Nb*: population at reduction, *NB*: population at bottleneck; *t_i_* : time of an event (expressed in generations ago) *DB*: duration of bottleneck; *DE*: duration of expansion.
