# Supplementary material for: Alternative glacial-interglacial refugia demographic hypotheses tested on Cephalocereus columna-trajani (Cactaceae) in the intertropical Mexican drylands
Source: PLoS One. 2017 Apr 20;12(4):e0175905. doi: 10.1371/journal.pone.0175905 (PMC5398652; doi:10.1371/journal.pone.0175905)
Supplement: S2 Table — Loadings of each variable in the first three principal components (PCi). Bold shown most important variables. (DOCX) [file pone.0175905.s004.docx]

**S2 Table**. **Principal component analysis of climatic variables.** Loadings of each variable in the first three principal components (PC_i_). Bold shown most important variables.

| Variable | PC1 | PC2 | PC3 |
| --- | --- | --- | --- |
| Bio1 | **-0.113** | **-0.239** | **0.376** |
| Bio2 | -0.075 | 0.001 | 0.100 |
| Bio3 | **0.152** | **-0.110** | -0.032 |
| Bio4 | -0.009 | -0.001 | 0.005 |
| Bio5 | -0.075 | -0.076 | -0.019 |
| Bio6 | 0.061 | **0.231** | -0.007 |
| Bio7 | **0.120** | **0.159** | -0.083 |
| Bio8 | -0.018 | 0.032 | -0.026 |
| Bio9 | -0.020 | 0.009 | 0.004 |
| Bio10 | **0.266** | -0.029 | **-0.243** |
| Bio11 | -0.080 | 0.101 | -0.091 |
| Bio12 | 0.001 | 0.001 | 0.001 |
| Bio13 | 0.001 | 0.003 | -0.002 |
| Bio14 | 0.002 | 0.000 | 0.004 |
| Bio15 | 0.001 | -0.003 | 0.003 |
| Bio16 | 0.000 | -0.003 | 0.000 |
| Bio17 | -0.002 | -0.001 | -0.004 |
| Bio18 | -0.001 | 0.001 | 0.000 |
| Bio19 | -0.004 | -0.001 | -0.001 |

Name of variables

**Bio01 Annual Temperature**

Bio02 Mean Monthly Temperature Range

**Bio03 Isothermality (*_100)**

Bio04 Temperature Seasonality (STD_*_100)]

Bio05 Maximum Temperature of Warmest Month]

**Bio06 Minimum Temperature of Coldest Month**

**Bio07 Temperature Annual Range**

Bio08 Mean Temperature of Wettest Quarter

**Bio09 Mean Temperature of Driest Quarter**

Bio10 Mean Temperature of Warmest Quarter

Bio11 Mean Temperature of Coldest Quarter

Bio12 Annual Precipitation

Bio13 Precipitation of Wettest Month

Bio14 Precipitation of Driest Month

Bio15 Precipitation Seasonality (CV)

Bio16 Precipitation of Wettest Quarter

Bio17 Precipitation of Driest Quarter

Bio18 Precipitation of Warmest Quarter

Bio19 Precipitation of Coldest Quarter
