# Supplementary material for: Alternative glacial-interglacial refugia demographic hypotheses tested on Cephalocereus columna-trajani (Cactaceae) in the intertropical Mexican drylands
Source: PLoS One. 2017 Apr 20;12(4):e0175905. doi: 10.1371/journal.pone.0175905 (PMC5398652; doi:10.1371/journal.pone.0175905)
Supplement: S3 Table — A) Values of different estimator in MANOVA. After obtaining a significant MANOVA, we test each variable with an ANOVA, and all bioclimatic condition were statistically different among all periods of time. F test value, DF1 = degree of freedom among groups; DF2 = degree of freedom of error; P-value. (DOCX) [file pone.0175905.s005.docx]

**S3 Table**. **Comparison of climatic variables among the four periods analysed.** A) Values of different estimator in MANOVA.

A) MANOVA

| Estimator | Observed Value | *F* | *DF1* | *DF2* | *P* |
| --- | --- | --- | --- | --- | --- |
| Wilks Lamnda | 0.0001 | 351.7 | 57 | 388 | <0.0001 |
| Hotelling-Lawley λ | 232.13 | 523.9 | 57 | 388 | <0.0001 |
| Pillai Trace | 2.91 | 231.7 | 57 | 388 | <0.0001 |
| Root of Roy | 169.35 | 1176.5 | 57 | 388 | <0.0001 |

After obtaining a significant MANOVA, we test each variable with an ANOVA, and all bioclimatic condition were statistically different among all periods of time, see below:

B) ANOVA

| Variable | *F* | *P* | DF1 | DF2 |
| --- | --- | --- | --- | --- |
| Annual Temperature | 30.679 | <0.0001 | 3 | 148 |
| Mean Monthly Temperature Range | 97.246 | < 0.0001 | 3 | 148 |
| Isothermality (*_100) | 289.536 | < 0.0001 | 3 | 148 |
| Temperature Seasonality (STD_*_100)] | 738.455 | < 0.0001 | 3 | 148 |
| Maximum Temperature of Warmest Month] | 27.155 | < 0.0001 | 3 | 148 |
| Minimum Temperature of Coldest Month | 61.534 | < 0.0001 | 3 | 148 |
| Temperature Annual Range | 832.652 | < 0.0001 | 3 | 148 |
| Mean Temperature of Wettest Quarter | 40.588 | < 0.0001 | 3 | 148 |
| Mean Temperature of Driest Quarter | 29.003 | < 0.0001 | 3 | 148 |
| Mean Temperature of Warmest Quarter | 32.466 | < 0.0001 | 3 | 148 |
| Mean Temperature of Coldest Quarter | 43.484 | < 0.0001 | 3 | 148 |
| Annual Precipitation | 5.661 | 0.001 | 3 | 148 |
| Precipitation of Wettest Month | 12.058 | < 0.0001 | 3 | 148 |
| Precipitation of Driest Month | 17.161 | < 0.0001 | 3 | 148 |
| Precipitation Seasonality (CV) | 16.533 | < 0.0001 | 3 | 148 |
| Precipitation of Wettest Quarter | 2.913 | 0.036 | 3 | 148 |
| Precipitation of Driest Quarter | 46.280 | < 0.0001 | 3 | 148 |
| Precipitation of Warmest Quarter | 42.807 | < 0.0001 | 3 | 148 |
| Precipitation of Coldest Quarter | 28.553 | < 0.0001 | 3 | 148 |

*F* test value, *DF1*= degree of freedom among groups; *DF2*= degree of freedom of error; *P*-value.
